# Case review analysis of operating room decisions to cancel surgery

**DOI:** 10.1186/1471-2482-14-47

**Published:** 2014-07-23

**Authors:** Ju-Hsin Chang, Ke-Wei Chen, Kuen-Bao Chen, Kin-Shing Poon, Shih-Kai Liu

**Affiliations:** 1 Department of Anesthesiology, China Medical University Hospital, China Medical University, 2 Yuh-Der Rd., Taichung 40447, Taiwan; 2 Division of Cardiology, Department of Internal Medicine, China Medical University Hospital, China Medical University, Taichung, Taiwan

**Keywords:** Preoperative care, Surgical procedures, Appointments and schedules

## Abstract

**Background:**

Cancellation of surgery close to scheduled time causes a waste of healthcare resources. The current study analyzes surgery cancellations occurring after the patient has been prepared for the operating room, in order to see whether improvements in the surgery planning process may reduce the number of cancellations.

**Methods:**

In a retrospective chart review of operating room surgery cancellations during the period from 2006 to 2011, cancellations were divided into the following categories: inadequate NPO; medical; surgical; system; airway; incomplete evaluation. The relative use of these reasons in relation to patient age and surgical department was then evaluated.

**Results:**

Forty-one percent of cancellations were for other than medical reasons. Among these, 17.7% were due to incomplete evaluation, and 8.2% were due to family issues. Sixty seven percent of cancelled cases eventually received surgery. The relative use of individual reasons for cancellation varied with patient age and surgical department. The difference between cancellations before and after anesthesia was dependent on the causes of cancellation, but not age, sex, ASA status, or follow-up procedures required.

**Conclusion:**

Almost half of the cancellations were not due to medical reasons, and these cancellations could be reduced by better administrative and surgical planning and better communication with the patient and/or his family.

## Background

Cancellations of surgical procedures can result in inefficient use of hospital resources and loss of hospital income. The most damaging cancellations with regard to inefficient and costly use of medical resources are cancellations that occur after the patient has been prepared for the operating room. Reasons for cancellations vary, depending on the patient population, the type of surgery, and the adequacy of hospital administrative procedures. For example, in the pediatric population scheduled for day surgery, but not other populations, 35 to 40% of cancellations were reported to be due to upper respiratory infections [1,2].

Many cancellations are either due to poor allocation of hospital facilities or poor prior evaluation of and communication with the patient. In a study of cancellations in a hospital that had increased its operating room capacity but not made any other changes, cancellations due to lack of post-operative bed space increased [3]. In a study of same day urological surgery, 77% of cancellations in the week before surgery were due to work-up or facility factors [4]. And in 2 studies of same day eye surgery, 20-40% of cancellations occurred because the patient did not show up on the day of surgery [5,6].

If the reasons for cancellations can be determined, procedures to avoid some of the cancellations can be put in place. Previous studies have focused on cancellations on the day of surgery. In contrast, the current study has a narrower focus and, through a retrospective chart review, examines and categorizes reasons for cancellation of surgery after the patient has been prepared for the operating room, in order to determine ways to reduce the number of such cancellations.

## Methods

This study was a retrospective chart review of all scheduled surgical procedures for the years 2006–2011 from the China Medical University Hospital. In these records, any surgery that was cancelled after the patient had already been prepared for the operating room was included in the study. The study was approved by the Institutional Review Board of China Medical University.

In our hospital system, every patient who enters the operation room (OR) is accompanied by his/her personal anesthetist and anesthesia technician(s), and cancellation of surgery is immediately recorded by quality assurance team members into a self-developed database connected to the hospital intranet. The information of all cases in this study was obtained from the hospital database containing the details of all operations performed. Data collection was done by independently designated personnel who were also responsible for the classification of each case. Further data retrieval and analysis were performed by one of the authors of this study, who also audited the input of data (including the classification and person responsible for input; therefore “inter-observer” differences were minimized).

The classification system used was developed to cover as many situations as possible and to separate “avoidable” from “unavoidable” reasons for cancellation. Reasons for cancelling surgeries were placed into the following 7 categories: inadequate NPO preparation, medical reasons, surgical reasons, system reasons, airway issues, family issues, and incomplete evaluation. (1) NPO preparation meant no oral food or fluid in the 12 hours previous to the surgery (2) Medical reasons included the following: highly elevated blood pressure; indications such as fever or unexpected infection; abscess formation outside the operating area; newly reported blood culture data; occurrence of an unstable hemodynamic change such as new onset arrhythmia or profound hypotension after anesthetic induction; and cardiac arrest. (3) Surgical reasons were a change by the surgeon of the surgical plan, usually due to a change in the patient’s clinical condition. This reason for cancellation usually did not result in increased anesthesiological risk, but led to a change in the operative approach and different follow-up care. (4) System reasons included the patient being sent to the operating room without notifying the doctor in charge, the wrong patient being sent to the operating room, and failure to prepare the necessary instruments. (5) Airway issues meant difficulty in maintaining the airway. (6) Family issues included the patient or family changing their previous decision (refusal of anesthesia or surgery), and no family member being present to sign the consent form. (7) Incomplete evaluation included incomplete pre-operative evaluation, the results of pre-operative evaluation not yet available, lack of awareness of the poor results in the pre-operative examination, and difference of opinion between the anesthesiologist performing the pre-operative evaluation and the anesthesiologist giving the anesthesia.

### Statistical analysis

The demographics and characteristics of patients were summarized as mean ± standard deviation and range (minimum, maximum) for patient age, and n(%) for other categorical variables. Causes to cancel surgery according to department or patient age were also summarized as n(%) and compared using Fisher’s exact test. All statistical assessments were two-tailed and considered significant at *P* < 0.05. All statistical analyses were performed using SPSS 15.0 statistics software (SPSS Inc, Chicago, IL, USA).

## Results

A total of 417 patients were included for evaluation and all patient records were collected from year 2006 to year 2011. There were 211 males (129 < 65 yrs and 82 ≧65 yrs) and 206 females (104 < 65 yrs and 102 ≧65 yrs). Among them, 34 patients (8.1%) were American Society of Anesthesiologists (ASA) physical status 1, 148 (35.5%) were ASA 2, 203 (48.7%) were ASA 3, 25 (6.0%) were ASA 4, and 7 (1.7%) were ASA 5. Patient demographics and characteristics are shown in Table 1. Cases cancelled as a percentage of cases scheduled for the different specialties are shown in Table 2.

**Table 1:** Patients’ demographics and characteristics were presented by year (N = 417)

**Variables**	**Total**
	**(n = 417)**
Age	
mean ± SD	56.9 ± 22.5
(minimum, maximum)	(1, 96)
Numbers of Age > 65 yrs	184 (44.1%)
Sex	
Male	211 (50.6%)
Female	206 (49.4%)
ASA physical status	
1	34 (8.1%)
2	148 (35.5%)
3	203 (48.7%)
4	25 (6.0%)
5	7 (1.7%)
Surgery cancelled after anesthesia^a^	
Yes	31 (12.7%)
No	213 (87.3%)
Cause of surgery cancellation	
Inadequate NPO before anesthesia	14 (3.4%)
Medical reason	246 (59.0%)
Surgical reason	36 (8.6%)
System reason	8 (1.9%)
Airway issue	5 (1.2%)
Family issue	34 (8.2%)
Incomplete evaluation	74 (17.7%)
Follow-up procedure^b^	
Received operation at a later time	277 (67.1%)
Did not receive operation	136 (32.9%)

Data are presented as mean ± SD with range (minimum, maximum) for age and n(%) for other variables.

^a^173 patients were missing.

^b^4 patients were missing.

**Table 2:** Cases cancelled as a percentage of scheduled cases

**Specialty**	**No. of scheduled cases**	**No. of cancelled cases**	**Percentage of cancelled cases (%)**
General Surgery	47,810	127	0.27
Gastrointestinal/Urology	19,611	80	0.41
Neurosurgery	15,376	28	0.18
Orthopedics	31,449	84	0.27
Gynecology	10,080	23	0.23
Trauma Surgery	16,458	39	0.24
Internal Medicine	55,437	36	0.06
**Total**	**196,221**	**417**	**0.21**

There were 246 (59%) cancellations due to medical reasons; 74 (17.7%) to incomplete evaluations; 36 (8.6%) to surgical reasons; 34 (8.2%) to family issues; 14 (3.4%) to inadequate NPO; 8 (1.9%) to system reasons, and 5 (1.2%) to airway issues (Table 3). For 31 patients (12.7%), the surgery was cancelled after general anesthesia had been induced.

**Table 3:** Summary of patients’ demographics, surgeon’s characteristics by cause to cancel surgery (N = 417)

	**Cause to cancel surgery**							
**Variables**	**NPO before anesthesia**	**Medical reason**	**Surgical reason**	**System reason**	**Airway issue**	**Family issue**	**Incomplete evaluation**	**P-value**
*N*	*14*	*246*	*36*	*8*	*5*	*34*	*74*	
Sex								0.250
Males	11 (80%)	121 (49%)	20 (56%)	5 (63%)	4 (80%)	16 (47%)	34 (46%)	
Females	3 (21%)	125 (51%)	16 (44%)	3 (38%)	1 (20%)	18 (53%)	40 (54%)	
Age								
<18 years	3 (21%)	25 (10%)	1 (3%)	3 (38%)	1 (20%)	0 (0%)	3 (4%)	0.018^*^
18 years ≦ age < 65 years	8 (58%)	116 (47%)	18 (50%)	3 (38%)	3 (60%)	13 (38%)	36 (49%)	
≧ 65 years	3 (21%)	105 (43%)	17 (47%)	2 (24%)	1 (20%)	21 (62%)	35 (47%)	
Visit pre-operative anesthesia OPD^a^								0.256
No	4 (30.8%)	82 (33.7%)	8 (21.6%)	0 (0%)	2 (40%)	7 (20%)	22 (29.7%)	
Yes	9 (69.2%)	161 (66.3%)	29 (78.4%)	7 (100%)	3 (60%)	28 (80%)	52 (70.3%)	
Surgeon department								0.049^*^
General Surgery	5 (35.7%)	74 (30.1%)	8 (22.2%)	4 (50.0%)	2 (40%)	8 (23.5%)	26 (35.1%)	
Gastrointestinal/Urology	4 (28.6%)	40 (16.3%)	16 (44.4%)	2 (25.0%)	0 (0%)	6 (17.6%)	12 (16.2%)	
Neurosurgery	0 (0%)	17 (6.9%)	2 (5.6%)	0 (0%)	1 (20%)	2 (5.9%)	6 (8.1%)	
Orthopedics	0 (0%)	51 (20.7%)	6 (16.7%)	1 (12.5%)	0 (0%)	8 (23.5%)	18 (24.3%)	
Gynecology	0 (0%)	16 (6.5%)	0 (0%)	0 (0%)	0 (0%)	2 (5.9%)	5 (6.8%)	
Trauma Surgery	2 (14.3%)	27 (11%)	1 (2.8%)	1 (12.5%)	2 (40.0%)	1 (3.0%)	5 (6.8%)	
Internal Medicine	2 (21.4%)	21 (8.5%)	3 (8.3%)	0 (0%)	0 (0%)	7 (20.6%)	2 (2.7%)	
Surgery cancelled after anesthesia^b^								
No	11 (100%)	132 (89%)	15 (68%)	2 (50%)	3 (60%)	19 (83%)	31 (100%)	0.001^*^
Yes	0 (0%)	16 (11%)	7 (32%)	2 (50%)	2 (40%)	4 (17%)	0 (0%)	
Follow-up procedure^c^								0.031^*^
Received operation	13 (92.9%)	157 (64.6%)	22 (61.1%)	5 (71.4%)	2 (40%)	20 (58.8%)	58 (78.4%)	
Did not receive operation	1 (7.1%)	86 (35.4%)	14 (38.9%)	2 (28.6%)	3 (60%)	14 (41.2%)	16 (21.6%)	

Results were summarized as n(%)and comparedusing Fisher’s exact test.

OPD, outpatient department.

^a^6 patients were missing.

^b^173 patients were missing.

^c^4 patients were missing.

*indicates significantly different (*P* < 0.05).

Follow-up of the cancellations showed that 277 (67.1%) of the cancelled operations were performed at a later time (that is, the patients still received the surgery, but after some further treatment). This information suggests that some of the reasons causing surgery cancellation could be amended or avoided, but that the cancelled surgery was ultimately necessary. Furthermore, 43 out of the 277 patients who had the operation at a later time did so with local anesthesia (that is, the surgeon decided to do local anesthesia independently, without involving the anesthesiologist). At the time of data collection, 136 patients (32.9%) did not have the cancelled operation at a later time (Table 1).

Table 3 summarizes the reasons for cancellation according to patient demographics and department. The distributions of causes for surgery cancellations were significantly different between the age groups of the patients, the surgeons’ departments, cancelled before or after anesthesia, and whether patients received follow-up procedures (All P-values <0.05; Table 3). The sex of the patients and visit to pre-operative anesthesia OPD were not associated with the causes.

Table 4 compares reasons for cancellation in patients who had already been anesthetized and those who had not yet been anesthetized. Between these two groups, there were no significant differences in terms of age, sex, ASA physical status, or whether the patients received follow-up procedures. The only significant difference was the cause of surgery cancellation (p < 0.001). For example 22.6% of cancellations after anesthesia, but only 7% of cancellations before anesthesia due to surgical reasons, and 0% of cancellations after anesthesia, but 14.6% of cancellations before anesthesia were due to incomplete evaluation.

**Table 4:** Differences in patient demographics and characteristics between patients with surgery cancelled before and after anesthesia (n = 244)

**Variables**	**Total**	**Surgery cancelled after anesthesia**	**Surgery cancelled before anesthesia**	**p-value**
	**(n = 244)**	**(n = 31)**	**(n = 213)**	
Age				
mean ± SD	57.4 ± 22.8	56.2 ± 25.3	57.6 ± 22.5	0.974
(minimum, maximum)	(1 to 96)	(1 to 85)	(1 to 96)	
Sex				0.093
Male	131 (53.7%)	21 (67.7%)	110 (51.6%)	
Female	113 (46.3%)	10 (32.3%)	103 (48.4%)	
ASA physical status				0.21
1	19 (7.8%)	2 (6.5%)	17 (8.0%)	
2	94 (38.5%)	11 (35.5%)	83 (39.0%)	
3	112 (45.9%)	13 (41.9%)	99 (46.5%)	
4	15 (6.1%)	3 (9.7%)	12 (5.6%)	
5	4 (1.6%)	2 (6.5%)	2 (0.9)	
Cause of surgery cancellation				<.001^*^
Inadequate NPO before anesthesia	11 (4.5%)	0 (0%)	11 (5.2%)	
Medical reason	148 (60.7%)	16 (51.6%)	132 (62.0%)	
Surgical reason	22 (9.0%)	7 (22.6%)	15 (7.0%)	
System reason	4 (1.6%)	2 (6.5%)	2 (0.9%)	
Airway issue	5 (2.0%)	2 (6.5%)	3 (1.4%)	
Family issue	23 (9.4%)	4 (13.0%)	19 (8.9%)	
Incomplete evaluation	31 (12.7%)	0 (0%)	31 (14.6%)	
Follow-up procedure				0.555
Received operation at a later time	161 (66.0%)	19 (61.3%)	253 (66.7%)	
Did not receive operation	83 (34.0%)	12 (38.7%)	71 (33.3%)	

Data are presented as mean ± SD with range (minimum, maximum) for age and n(%) for other variables by patients with or without cancelling surgery after anesthesia.

Difference between groups were compared using Mann–Whitney U test for age due to age was not normally distributed; and Pearson Chi-sqaure test or Fisher’s exact if cell numbers less than five for other categorical variables.

^*^P < 0.05, indicates significantly different between groups.

## Discussion

This retrospective chart review of reasons that surgery was cancelled after the patient had been prepared for surgery showed that the most common reason for cancellation was medical, but that many cancellations were due to situations that could be prevented with changes in scheduling, administrative, and communications procedures.

Incomplete pre-operative evaluation, systemic problems, and insufficient NPO are due to administrative errors that should be corrected. Some reasons like family causes should be avoided by full discussion and communication with the families before the patients arrive at the OR. And although a change in medical condition leading to cancellation was the main cause of cancellations and would seem to be an uncontrollable factor, reinforcement of communication between departments might prevent some of these cancellations.

Cancellation after the patient and operating room have been prepared for surgery causes definite financial loss to the institution, while cancellations at earlier times may cause less to no real financial loss. Also, some reasons for earlier cancellations are not pertinent to very late cancellations. Therefore, it is not appropriate to directly compare the reasons and frequency for cancellation in this report to other reports focusing on cancellations at much earlier time periods. Few reports on same day cancellation of in-patient surgery have been published. Rates for same day cancellations have been reported to be 12-24% [3], but some of these rates are for day and not in-patient surgery. For same day cancellation of in-patient surgery, Schofield [7] reported a 12% cancellation rate and that 58% of these cancellations were due to OR overrun, lack of availability of post-operative beds, or procedural errors, and only 17% were due to changes in patient status. In a similar study, Pollard [8] reported a 13% cancellation rate and that the most common reason for cancellation was administrative problems, such as insufficient OR time. In reports from Nigeria, India, and Saudi Arabia, unavailability of surgeon, OR, or ICU beds were major reasons for same day cancellations, and the surgeon’s underestimation of OR time was an important cause of OR unavailability [9-12]. However, in a report on pediatric surgery in Australia, the contribution of these causes to same day cancellations was minimal [13].

Perroca et al. [14], n the study most comparable to this study, reported 53% of cancellations that occurred after the OR had been prepared for surgery were patient-related. Most of the cancellations (29%) were due to the patient’s medical condition, and 24% were due to organizational problems, such as lack of ICU beds. In our study, 59% of cancellations were due to medical reasons. Our data showing 12.7% of OR cancellations made after anesthesia is also similar to the 9.1% of OR cancellations made during surgery reported by Perroca et al. [14].

Several studies have reported on specific methods used in attempt to reduce surgery cancellations. At one hospital, the single change of increasing operating room capacity did not decrease cancellations because post-operative bed capacity was not increased at the same time [3]. At another hospital, instituting outpatient preoperative evaluation of patients did decrease cancellations [15]. And an analysis of the effect of scheduling cases with known predictors of a high rate of cancellation to the last operative period of the day concluded that using this means of reducing cancellations was impractical [16].

To relate our results to those of others, it is useful to put them in the context of the health care system in Taiwan. Health care is Taiwan is operated by the government with relatively low co-payments from patients [17,18]. Therefore, many tests and surgeries have become quite cheap and are over-prescribed. In addition, with the high population density and high hospital density in Taiwan, medical care is highly accessible. And Taiwanese people tend to feel physiologically more secure if more tests are done, more medications ordered, and surgery performed when possible (not when necessary). Occasionally there are even surgeries cancelled because the patients wanted to schedule the surgery as soon as possible to the point that the pre-surgery test results were not yet available. These situations may provide useful information for other countries promoting public health care (for example, the US).

The current study has a number of limitations. (1) It is a retrospective study. (2) It is difficult to separate reasons for cancellations into clear-cut, discrete categories, for some cancellation reasons might fit in more than one category and some cancellations might be described differently depending on the individual who recorded them. (3) Further data analyses are needed to determine whether there were more cancellations due to inadequate preparation in “emergency” vs. in “non-emergency” operations. (4) Although we could evaluate group differences in causes for cancellation, we could not do statistics on differences between groups in each individual cause because of the small sample size of some of the individual causes. In the future, we are interested in examining the time lost between surgery cancellation, vacation of the occupied space, and the time and manpower required to prepare for the next scheduled surgery, and estimating the financial costs of the cancellations.

## Conclusions

In conclusion, in this study analyzing cases of in-patient surgery cancellations after the patient had been prepared for the operating room, the majority of cancellations were due to unforeseen changes in patient status, but many were due to oversights or inadequacies in procedures that could be improved.

## Abbreviations

OR: Operating room; NPO: Nil per os, nothing by mouth; SD: Standard deviation; OPD: Outpatient department.

## Competing interests

The authors declare that they have no competing interests.

## Authors’ contributions

JHC drafted the manuscript and analyzed data. KWC participated in the sequence alignment and drafted the manuscript. KBC participated in the sequence alignment and drafted the manuscript. KSP participated in the sequence alignment and drafted the manuscript. SKL participated in the study design and final manuscript. All authors read and approved the final manuscript.

## Pre-publication history

The pre-publication history for this paper can be accessed here:

http://www.biomedcentral.com/1471-2482/14/47/prepub
